# Altered RBP1 Gene Expression Impacts Epithelial Cell Retinoic Acid, Proliferation, and Microenvironment

**DOI:** 10.3390/cells11050792

**Published:** 2022-02-24

**Authors:** Jianshi Yu, Mariarita Perri, Jace W. Jones, Keely Pierzchalski, Natalia Ceaicovscaia, Erika Cione, Maureen A. Kane

**Affiliations:** 1 Department of Pharmaceutical Sciences, University of Maryland School of Pharmacy, Baltimore, MD 21201, USA; jyu@rx.umaryland.edu (J.Y.); mperri82@gmail.com (M.P.); jjones@rx.umaryland.edu (J.W.J.); keelski1@gmail.com (K.P.); nceaic1@gmail.com (N.C.); 2 Department of Pharmacy, Health and Nutritional Sciences, University of Calabria, Ed. Polifunzionale, I-87036 Arcavacata di Rende, CS, Italy; erika.cione@unical.it

**Keywords:** breast cancer, Retinol binding protein 1 (RBP1), microenvironment, proliferation, all-trans retinoic acid (atRA), tumorigenesis, epithelial cell, fibroblast, mass spectrometry

## Abstract

Vitamin A is an essential diet-derived nutrient that has biological activity affected through an active metabolite, all-trans retinoic acid (atRA). Retinol-binding protein type 1 (RBP1) is an intracellular chaperone that binds retinol and retinal with high affinity, protects retinoids from non-specific oxidation, and delivers retinoids to specific enzymes to facilitate biosynthesis of RA. RBP1 expression is reduced in many of the most prevalent cancers, including breast cancer. Here, we sought to understand the relationship between RBP1 expression and atRA biosynthesis in mammary epithelial cells, as well as RBP1 expression and atRA levels in human mammary tissue. We additionally aimed to investigate the impact of RBP1 expression and atRA on the microenvironment as well as the potential for therapeutic restoration of RBP1 expression and endogenous atRA production. Using human mammary ductal carcinoma samples and a series of mammary epithelial cell lines representing different stages of tumorigenesis, we investigated the relationship between RBP1 expression as determined by QPCR and atRA via direct liquid chromatography-multistage-tandem mass spectrometry-based quantification. The functional effect of RBP1 expression and atRA in epithelial cells was investigated via the expression of direct atRA targets using QPCR, proliferation using Ki-67 staining, and collagen deposition via picrosirius red staining. We also investigated the atRA content of stromal cells co-cultured with normal and tumorigenic epithelial cells. Results show that RBP1 and atRA are reduced in mammary tumor tissue and tumorigenic epithelial cell lines. Knock down of RBP1 expression using shRNA or overexpression of RBP1 supported a direct relationship between RBP1 expression with atRA. Increases in cellular atRA were able to activate atRA direct targets, inhibit proliferation and inhibit collagen deposition in epithelial cell lines. Conditions encountered in tumor microenvironments, including low glucose and hypoxia, were able to reduce RBP1 expression and atRA. Treatment with either RARα agonist AM580 or demethylating agent Decitabine were able to increase RBP1 expression and atRA. Cellular content of neighboring fibroblasts correlated with the RA producing capacity of epithelial cells in co-culture. This work establishes a direct relationship between RBP1 expression and atRA, which is maintained when RBP1 expression is restored therapeutically. The results demonstrate diseases with reduced RBP1 could potentially benefit from therapeutics that restore RBP1 expression and endogenous atRA.

## 1. Introduction

Vitamin A is an essential diet-derived nutrient that has biological activity affected through an active metabolite, retinoic acid (RA) [1,2]. RA is a master regulator of proliferation, differentiation, and apoptosis [3]. RA has a number of geometric isomers with differing biological activity. All-*trans*-retinoic acid (atRA) carries out the vast majority of biological activity by binding to nuclear receptors, including several isoforms of retinoic acid receptor (RAR α, β, γ) and peroxisome proliferator-activated receptor (PPAR β/δ) with high affinity to initiate transcription [4,5]. A number of non-genomic actions have also been reported for atRA [6,7]. 9-*cis*-retinoic acid (9cRA), binds with high affinity to RAR and retinoid X receptor (RXR) [8], but detection has been limited in vivo to the pancreas where it has been shown to regulate glucose sensing [9,10,11]. 13-*cis*-retinoic acid (13cRA) and 9,13-di-*cis*-retinoic acid (9,13dcRA) have both been detected in vivo, but do not bind to nuclear receptors with high affinity [4,12,13]. Each of those ligand-activated nuclear receptors has distinct roles in physiology and is dependent upon the availability of their high-affinity ligand [14]. atRA homeostasis is regulated by a series of enzymes and chaperones [1,2,15,16].

Retinol-binding protein type 1 (RBP1) is an intracellular chaperone that binds retinol and retinal with high affinity, protects retinoids from non-specific oxidation, and delivers retinoids to specific enzymes [2,15,16]. RBP1-bound retinol serves as substrate for retinol dehydrogenases (RDHs), which catalyze the first reversible and rate-limiting step in atRA biosynthesis [2]. RBP1-bound retinal serves as the substrate for retinal dehydrogenases (RALDHs), which catalyze the second irreversible step in atRA biosynthesis [2]. RBP1-bound retinoid is the preferred substrate for both RDH and RALDH in atRA biosynthesis with more efficient enzymatic activity than free substrate [1].

RBP1 expression is reduced in many of the most prevalent cancers including breast, prostate, lung, colon and rectal, melanoma, bladder, non-Hodgkin lymphoma, leukemia, endometrial, and pancreatic cancer [17,18,19,20,21,22,23,24,25,26]. Loss of RBP1 was reported to be an early event in cancer and RBP1 expression was reduced in normal tissue adjacent to tumors with reduced RBP1 [17,26]. We have previously observed a reduction in atRA in *Rbp1^-/-^* mouse tissues including mammary, heart, lung, endometrium, testis, and liver [27,28,29,30,31]. *Rbp1^-/-^* mammary exhibited epithelial cell hyperplasia and increased collagen characteristic of the dysfunction in tissue homeostasis that precedes tumor formation [31]. Epithelial cells produce atRA, express RBP1, and are important to mammary tumorigenesis [32,33,34,35]. It is presumed that loss of epithelial cell RBP1 results in reduced atRA, but this relationship between RBP1 expression and atRA levels has not been rigorously characterized through direct measurement of active metabolite atRA. Here, we sought to understand the relationship between RBP1 expression and atRA biosynthesis in mammary epithelial cells, as well as RBP1 expression and atRA levels in mammary tissue. We additionally aimed to investigate the impact of RBP1 expression and atRA on the microenvironment, as well as the potential for therapeutic restoration of RBP1 expression and endogenous atRA production.

## 2. Materials and Methods

### 2.1. Cell Culture

MCF-7 and MDA-MB-231 cell lines were obtained from American Type Culture Collection (ATCC) and maintained in Dulbecco’s Modified Eagle Medium (DMEM) supplemented with 10% Fetal Bovine Serum (FBS). MCF-10F and MCF-10-2A cell lines were obtained from ATCC and maintained in DMEM/F-12 media supplemented with cholera toxin, hydrocortisone, insulin, EGF (Epidermal Growth Factor) and 5% horse serum. Human mammary epithelial cells (HMEC) were generously provided by Dr. M. Stampfer from Lawrence Berkeley National Laboratory and maintained in mammary epithelial cell basal medium (MEBM), supplemented with Mammary Epithelial Cell Growth Medium (MEGM) kit (Lonza) and 1 ng/mL cholera toxin. The BJ-5ta cell line was generously provided by Dr. Anna E. Maciag from SAIC-Frederick, Inc. (Frederick, MD, USA), NCI-Frederick. All cells were grown at 37 °C and 5% CO_2_ atmosphere.

The HMEC is a primary normal human mammary epithelial cell line. The MCF-10F and MCF-10-2A cell lines are immortal, non-tumorigenic epithelial cell lines. The MCF-7 cell line is a breast cancer cell line with a low invasive phenotype in vitro. The MDA-MB-231 cell line is breast cancer cell lines with a highly invasive phenotype in vitro. BJ-5ta cell is an immortalized human fibroblast cell line.

Fibroblast and epithelial cell co-culture was conducted using the direct contact method. Epithelial cells and fibroblast cells were co-cultured in the same well in a ratio of 1:3 (epithelial:fibroblast). Co-cultured epithelial cells and fibroblast cells were detached by TrypLE Express (Invitrogen, Waltham, MA, USA) according to the product instructions. Cells were spun down and re-suspended in an isolation buffer, followed by labeling of the cells with biotinylated Ep-CAM antibody (Biolegend, San Diego, CA, USA), according to the product instructions. Cells were incubated 10 min in a cold room, followed by washing of the cells by adding an isolation buffer, and subsequent spin down of the cells. Cells were then re-suspended in an isolation buffer. Dynabeads Biotin Binder (Invitrogen) was added, and the cells were incubated for another 30 min in a cold room, after which the epithelial cells and fibroblast cells were magnetically separated. Fibroblast cells used in the co-culture experiment were BJ-5ta. Epithelial cells used in the co-culture experiment were MCF-7 cells.

### 2.2. Human Tissue Samples

Human tissues were obtained through the Stuart and Marlene Greenebaum Cancer Center Tissue Bank, as selected by a breast pathologist. Samples were de-identified and only patient demographics and diagnosis of ductal carcinoma (DCA) were used as determinants for this study. Normal tissues from reduction mammoplasty served as the control for DCA samples. Samples selected for atRA analysis were DCA with reduced RBP1 expression.

### 2.3. Preparation of All-Trans Retinol Bound to Human Retinol-Binding Protein 4 (holoRBP4)

holoRBP4 was prepared by a method adopted from Xie et al. [36]. Briefly, BL21 (λ*DE3*) cells containing a human *RBP4* expression plasmid were grown in LB kanamycin (50 μg/mL) broth to O.D. 0.6. Isopropyl b-D-thiogalactoside (IPTG) was added to 1 mM to induce protein expression for 4 h. Cells were spun down and collected as the bacteria pellet. The bacteria pellet was resuspended in Bacterial Protein Extraction Reagent (B-PER) buffer (Thermo Fisher Scientific, Waltham, MA, USA). Cells were agitated in a rotating shaker at room temperature for 20 min, after which bacterial cells were lysed. The lysed cells were centrifuged at 15,000 rpm at 4 °C followed by removal of the supernatant. The pellet was resuspended in buffer 1 (100 mM Tris HCl, 300 mM NaCl, 5 mM imidazole, 8 M urea, 5% *v*/*v* glycerol, pH 7.4). After agitation in a rotating shaker at room temperature for 30 min, samples were centrifuged at 7000 rpm at room temperature. The supernatant (denatured human RBP4 protein in buffer 1) was applied onto a Ni Sepharose^TM^ 6 Fast Flow (Cytiva, Marlborough, MA, USA, Cat# 17-5318-01) column. Denatured human RBP4 protein was eluted by buffer 2 (100 mM Tris HCl, 300 mM NaCl, 250 mM imidazole, 8 M urea, 5% *v*/*v* glycerol, pH 7.4). 10 mM DTT was added to the purified denatured RBP4 solution. The reduced purified denatured RBP4 solution was mixed with at least ten-fold excess of free all-*trans* retinol in ethanol and redox-refolding buffer (0.3 mM cystine, 3 mM cysteine, 1 mM EDTA, 25 mM Tris HCl, 10 mM DTT, pH 9.0) at 4 °C with a vigorous vortex to refold for at least 5 h. The refolded holoRBP4 was subjected to dialysis to remove urea and excess free retinol. The holoRBP4 was concentrated by Amicon ultra centrifugal filter units (MilliporeSigma, Burlington, MA, USA, Ultra-15, pore size 10 KDa NMWCO, Millipore, UFC901008) and changed to phosphate-buffered saline (PBS) buffer. The concentration was determined by UV absorbance at 280 nm, ε = 46,400 [37]. A typical dual peak UV spectrum (280 nm and 330 nm) was observed.

### 2.4. Decitabine (5-Aza-2′-deoxycytidine) Treatment

Cells were treated with 10 μM Decitabine (Sigma-Aldrich, St. Louis, MO, USA, 5-Aza-2′-deoxycytidine) for 3 days as adapted from Kastl et al. [38]. Decitabine was replenished every 24 h. Demethylated cell lines were incubated with and without 2 μM holoRBP4. Decitabine was chosen because treatment of MCF-7 and MDA-231 cells reduced methylation [38].

### 2.5. Treatment of Cells in Culture to Mimic Tumor Microenvironmental Factors

MCF-10-2A cell were grown in various conditions to mimic aspects of the tumor microenvironment in a cell culture condition, including low glucose and hypoxia.

Low glucose: Normal culture media contains 4.5 g/L glucose, which we defined as the “high” glucose condition. We defined the “low” glucose culture condition as 1.0 g/L glucose. MCF-10-2A cells were cultured in the low glucose condition for 24 h then, where indicated, were cultured with the addition of 1 μM MG132 (Sigma, carbobenzoxy-Leu-Leu-leucinal) for 24 h. MG132 is a potent, reversible, and cell-permeable proteasome inhibitor, previously used in cell culture and used here to assess the contribution of protein degradation [39].

Hypoxia: MCF-10-2A cells were cultured with 1 mM dimethyloxalyl glycine (DMOG) (Sigma, CAS 89464-63-1) or 150 μM Cobalt (II) chloride hexahydrate (CoCl_2_) (Sigma) for 24 h and then, where indicated, were cultured with the addition 1 μM MG132 for 24 h. Cells were grown according to the described conditions and then treated with 2 μM holoRBP4. Treatment concentrations and treatment times were adapted from the literature [39,40,41,42,43,44,45,46]. DMOG is cell-permeable and a competitive inhibitor of HIF-hydroxylases, thereby enabling stabilization and heterodimerization of HIF-1α, which has been extensively applied as a tool to mimic hypoxia [40,41,42,43,44,47]; CoCl_2_ is a commonly used model to mimic hypoxia, where CoCl_2_-induced chemical hypoxia stabilizes hypoxia inducible factors 1α and 2α under normoxic conditions [43,45,46,48]; and MG132 is a potent, reversible, and cell-permeable proteasome inhibitor previously used in cell culture and used here to assess the contribution of protein degradation [39].

### 2.6. RARα Agonist AM580 in Culture Condition

MCF-7 and MDA-MB-231 cell line were treated with 200 nM AM580 (Sigma, RARα agonist) for 48 h adapted from Bosch et al. [49]. Then, cell lines were incubated with 2 μM holoRBP4 for 3 h. AM580 is an RARα agonist that has been shown to increase the expression of RBP1 [49,50].

### 2.7. Retinoids Extraction and Quantification

For retinoid quantification, 0.1 million cells were seeded per well per 1 mL in a 12 well plate. Cells were grown in different conditions and treated with or without holoRBP4 for the period of time indicated. After incubation, 0.8 mL culture media was collected as the media sample for analysis, followed by removal of the remaining media. Lysis of cells was effected with addition of 0.3 mL Radioimmunoprecipitation assay buffer (RIPA buffer), followed by collection of all the lysate as the cell sample for analysis. Extraction and analysis were performed as previously reported with minor modification [13,27]. Ten microliters of internal standard (acitretin or 4,4-dimethyl-RA in acetonitrile) was added to each sample. Then, 1 mL of 0.025 M KOH in ethanol was added to the sample (800 μL cell culture media sample or 300 μL cell lysate sample). The aqueous phase was extracted with 5 mL of hexane. The organic phase containing nonpolar retinoids (retinol and retinyl ester(s)) was transferred to a new glass tube. Then, 4 M HCl (65 μL) was added to the aqueous phase, and polar retinoids (RA) were extracted by 5 mL hexane. Organic phases were removed under nitrogen with gentle heating at ~25–30 °C in a water bath (Organomation Associates Inc. model N-EVAP 112, Berlin, MA, USA). RA extracts were resuspended in 60 μL of acetonitrile. atRA produced by epithelial cells was quantified by liquid chromatography-multiple reaction monitoring cubed (LC-MRM^3^), using a Nexera UFLC liquid chromatography system (Shimadzu, Columbia, MD, USA) coupled with an 65,500 Qtrap hybrid triple quadrupole mass spectrometer (Sciex, Redwood City, CA, USA). LC-MRM^3^ is a liquid chromatography-multistage-tandem mass spectrometry method, where a precursor ion is fragmented, followed by an additional fragmentation of the product ion, where the second-generation fragment ion is used for quantification. MRM^3^, also referred to as MS^3^ or MS/MS/MS, offers additional selectivity to remove interferences and further lower detection limits in biological matrices. We have published the methodology describing this liquid chromatography-multistage-tandem mass spectrometry for quantification of atRA previously [27].

### 2.8. Total RNA Isolation and Q-PCR

Next, 3 × 10^5^ cells were seeded in each well of a 6-well plate. After treatment, total RNA was isolated using a RNeasy kit (QIAGEN, Germantown, MD, USA). Total RNA from 100 mg tissue was also isolated in this manner. RNA concentration was determined by Take3 plates on an Eon plate reader (BioTek, Winooski, VT, USA). 2 μg RNA was reverse transcribed to cDNA by High-Capacity cDNA Reverse Transcription Kit (Invitrogen). Gene expression was quantified by the TaqMan Gene expression assay (Invitrogen) and normalized to β-actin. Fold change was determined by Comparative Ct method.

### 2.9. Overexpression RBP1 in MCF-7 and MDA-MB-231 Cell Lines

Cells were cultured in DMEM media supplemented with 10% FBS. Transient or stable transfections of cells with mouse *Rbp1* gene plasmid were performed with an optimal ratio of DNAs and Lipofectamine 2000. Stable cell lines were isolated with Geneticin selection. After Geneticin selection, the cells were seeded into 12 well plate at 0.1 million cells per well per ml growth media for RA extraction or 6 well plate at 0.3 million cells per well for RNA isolation and gene expression. The *mRbp1* plasmid used in this study was a gift of Dr. Eduardo Farias [33].

### 2.10. Knock-down RBP1 in MCF-10-2A Cell Line by shRNA

We knocked down the expression of the human *RBP1* gene in the MCF-10-2A cell line using the TRC human shRNA clones. shRNA plasmid DNA was transfected by Lipofectamine 2000 (Invitrogen) into MCF-10-2A. Then, 24 h later, cells expressing shRNA were selected with puromycin for 2–3 weeks of gene expression and RA production analysis. The shRNA plasmids used in this study were MISSION^®^ TRC shRNA TRCN0000059975, MISSION^®^ TRC shRNA TRCN0000059976, MISSION^®^ TRC shRNA TRCN0000059977, MISSION^®^ TRC shRNA TRCN0000299258, MISSION^®^ TRC shRNA TRCN0000299259 and MISSION^®^ TRC shRNA TRCN0000299334 (Sigma-Aldrich, St. Louis, MO, USA).

### 2.11. Immunofluorescence Staining and Quantification for Ki-67

Ki-67 staining as a measure of cell proliferation was performed using a method adapted from previous studies in MCF-7 cells [51,52]. Here, MCF-7 and two independent stable overexpression *RBP1* clones were seeded in the Nunc™ Lab-Tek™ Chamber Slide System. Cells were incubated with 2 μM holoRBP4 for 3–7 days. Cell culture media was removed, and cells were fixed by 4% paraformaldehyde in PBS. After permeabilization and blocking, Ki-67 antibodies (Thermo Fisher Scientific, Catalog # PA5-19462) were added and incubated overnight in a cold room. Alexa Fluo 549 conjugated secondary antibodies were added for 1 h at room temperature. Slide was mounted by ProLong^®^ Gold Antifade Reagent with 4′,6-Diamidino-2-phenylindole (DAPI) (Thermo Fisher). Immunofluorescence images were taken using an AMG EVOS xl f1 microscope (Thermo Fisher Scientific). Ki-67 staining quantification was performed using ImageJ software FIJI distribution. First, we defined the nucleus as Regions of Interest (ROI) using the DAPI staining channel (blue) and then applied this ROI to the Ki-67 staining channel (red). The intensity of the ROIs was measured in the Ki-67 staining channel (red). The intensity was corrected by subtracting background intensity. The background reading was obtained from the average intensity outside the ROIs.

### 2.12. Collagen Staining and Quantification by Picrosirius Red in Cell Line Model

MCF-7 and two individual stable overexpression *RBP1* clones were seeded to 12 well plates. Culture media was removed after 18 h of incubation and the cells were washed once with ice-cold PBS. Cells were fixed with 10% neutral buffered formalin overnight. Formalin was removed and the cells were washed with PBS once. Then, 0.2 mL of 0.1% Sirius red in saturated picric acid solution was added to each well to stain the cells for 1 h at room temperature, followed by washing of the well twice with water and then drying of the plate. Images were taken with an AMG EVOS xl microscope (Thermo Fisher Scientific). For quantification, Sirius red was extracted with 0.5 mL of a mixture of 0.1 M NaOH in absolute methanol (1:1 *v*:*v*). The absorbance of the colored eluent was measured in an Eon plate reader (BioTek) at 540 nm. Data were normalized to the cell number and compared to parent cell lines.

### 2.13. Statistical Analysis

Statistics were performed with the GraphPad Prism software. Data are shown as mean ± SD, *n* = 3 or indicated otherwise. For a two-group comparison, an unpaired parametric two-tailed student t-test was used. For comparison of three or four groups, if each group compared to the control, then an ordinary one-way ANOVA analysis followed by Dunnett’s test was used. For comparison of three or four groups, if each group compared to every other group, then ordinary one-way ANOVA analysis followed by Tukey’s multiple comparison test was used. 

## 3. Results

### 3.1. RBP1 and atRA Are Reduced in Mammary Tumor Tissue and Tumorigenic Epithelial Cell Lines

**Figure 1:**
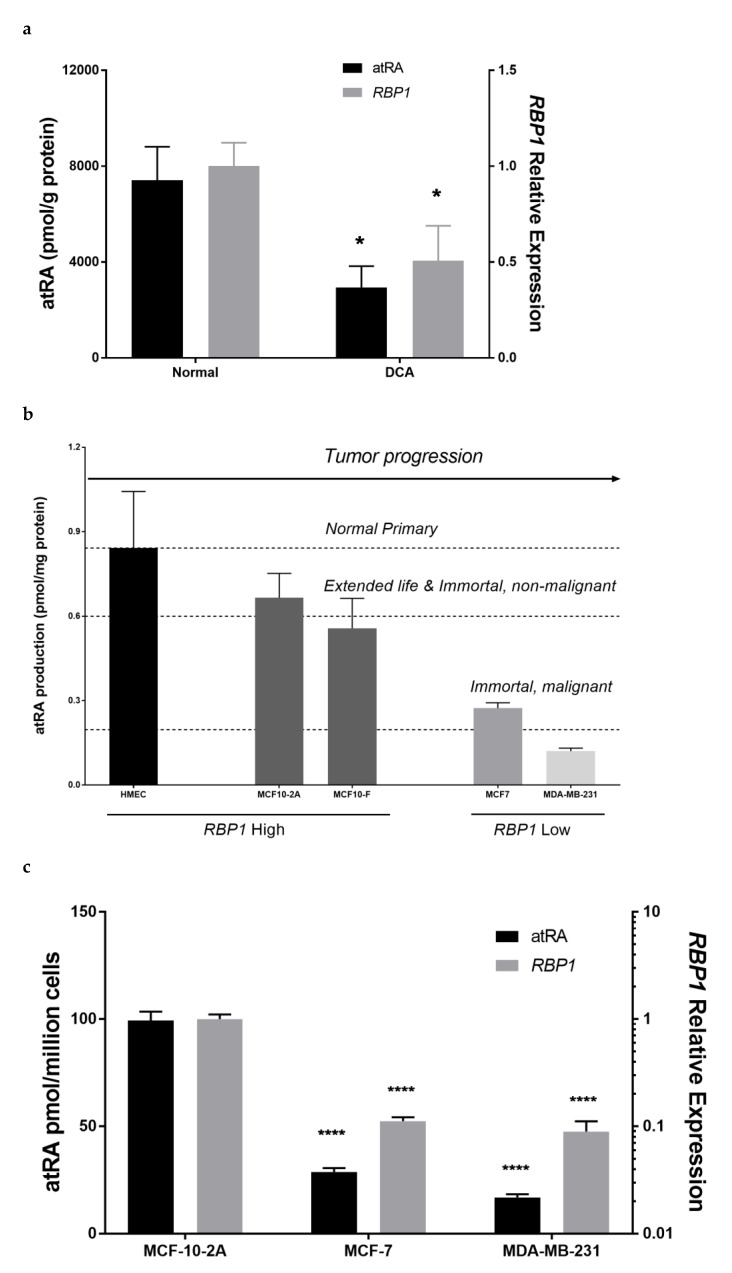

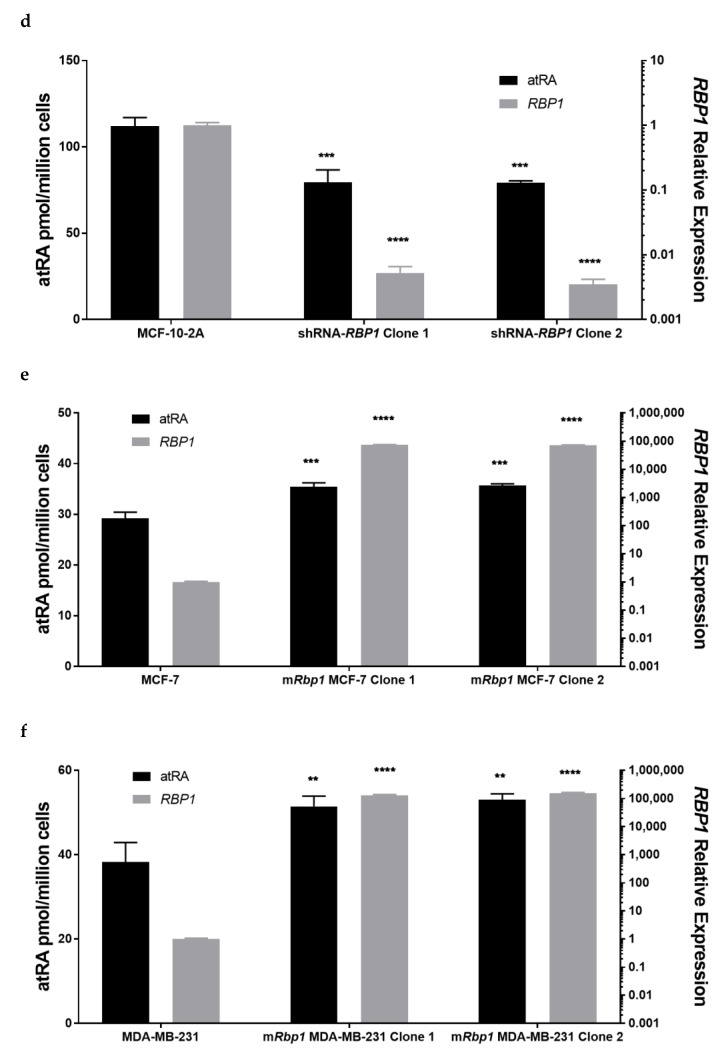
RBP1 expression has a direct relationship with atRA. (**a**) atRA was reduced in human mammary tissue with reduced RBP1 expression. Human ductal carcinoma (DCA) tissue or normal breast tissue from reduction mammoplasty; data were mean ± SD, *n* = 7. (**b**) Human mammary epithelial cell lines had reduced atRA as a function of tumorigenesis. Endogenous atRA in cell lines ranging from human primary mammary epithelial cells to invasive malignant breast cancer cell lines showed atRA decreases as a function of tumor progression. Cell lines include human mammary epithelial cells (HMEC), MCF-10-2A, MCF-10F, MCF-7 and MDA-MB-231 cells. Data were mean ± SD, *n* = 3. (**c**) Tumorigenic cell lines had reduced RBP1 expression and reduced atRA production. MCF-10-2A (normal), MCF-7 (tumorigenic) and MDA-MB-231 (tumorigenic) cells; data were mean ± SD, *n* = 3. (**d**) Knockdown of *RBP1* expression in normal MCF-10-2A resulted in reduced atRA production. Stable shRNA-*RBP1* knock down clones and parent MCF-10-2A cells; data were mean ± SD, *n* = 3. (**e**) Overexpression of *RBP1* in MCF-7 cells increased atRA production. Stable overexpression *mRbp1* clones and parent MCF-7 cells data were mean ± SD, *n* = 3. (**f**) Overexpression of RBP1 in MDA-MB-231 cells increased atRA production. Stable overexpression m*Rbp1* clones and parent MDA-MB-231 cells; data were mean ± SD, *n* = 3. In tissue, atRA was quantified with LC-MRM^3^ (left *y*-axis) and *RBP1* gene expression was quantified by QPCR normalized to beta-actin (right *y*-axis). In cell lines, cells were treated with holoRBP4 followed by atRA quantification with LC-MRM^3^ (left *y*-axis) and *RBP1* gene expression was quantified by QPCR normalized to beta-actin (right *y*-axis). Statistical analysis: Figure 1a, unpaired parametric two-tailed student t test. * *p* ≤ 0.05. Figure 1c–f, ordinary one-way ANOVA analysis corrected for multiple comparisons using Dunnett’s test (with comparison to the respective control in each panel). The ANOVA result summary were as follows: Figure 1c, atRA, F(2, 6) = 786.1, **** *p* < 0.0001, *RBP1*, F(2, 6) = 213.8, **** *p* < 0.0001; Figure 1d, atRA, F(2, 6) = 41.34, *** *p* = 0.0003, *RBP1*, F(2, 6) = 275.7, **** *p* < 0.0001; Figure 1e, atRA, F(2, 6) = 55.32, *** *p* = 0.0001, *RBP1*, F(2, 6) = 3899, ****p < 0.0001; Figure 1f, atRA, F(2, 6) = 20.13, ** *p* = 0.0022, *RBP1*, F(2, 6) = 301.9, **** *p* < 0.0001.

RBP1 expression is reduced in numerous common cancers, including breast cancer [17,18,19,20,21,22,23,24,25,26]. In order to determine the relationship between RBP1 expression and atRA levels in human mammary tissue, we quantified endogenous atRA using a liquid chromatography–multistage tandem mass spectrometry method, LC-MRM^3^, and quantified RBP1 expression using quantitative real-time PCR (QPCR) in human breast ductal carcinoma tissue and normal tissue from reduction mammoplasty. Endogenous atRA was reduced to 40% of normal control in ductal carcinoma samples that had 50% reduced *RBP1* expression (Figure 1a). To determine if RA production in human mammary epithelial cell lines had a relationship with carcinogenesis, we measured the endogenous atRA in a series of cell lines, ranging from human primary mammary epithelial cells to malignant breast cancer cell lines, including normal human primary mammary epithelial cells (HMEC); immortal, non-tumorigenic MCF-10-2A and MCF-10-F; and immortal, malignant MCF-7 and MDA-MB-231 cells (Figure 1b). Normal cell lines (HMEC, MCF-10) produced more atRA than tumorigenic cell lines (MCF-7, MDA-MB-231), showing that, in epithelial cell lines that represent tumor progression, atRA biosynthesis decreases as a function of tumorigenesis, according to one-way ANOVA analysis comparing the various cell lines to HMEC to the control (*p* < 0.001) (Figure 1b).

### 3.2. Altered RBP1 Expression Has a Direct Relationship with Altered RA Biosynthesis

To determine the relationship between RBP1 expression and atRA production in human mammary epithelial cell lines, we quantified RBP1 expression and atRA production in normal MCF-10-2A and tumorigenic MCF-7 and tumorigenic MDA-MB-231 cells (Figure 1c). Cancer cell lines MCF-7 and MDA-MB-231 had lower RBP1 expression and produced less atRA as compared to normal MCF-10-2a cells. We further manipulated RBP1 expression via silencing or overexpression and quantified atRA production (Figure 1d–f). In MCF-10-2A cells that had higher atRA production and RBP1 expression (as compared to tumorigenic MCF-7 and MDA-MB-231), we used shRNA to knock-down RBP1 expression and selected two independent clones. The production of atRA in those 2 knock-down clones were decreased (Figure 1d). In tumorigenic MCF-7 and MDA-MB-231 that have lower atRA production and RBP1 expression (compared to normal MCF-10-2a), we overexpressed RBP1 and selected two independent clones (Figure 1e,f). Increased RBP1 expression resulted in increased atRA production in both MCF-7 (Figure 1e) and MDA-MB-231 (Figure 1f).

### 3.3. atRA Produced from Physiological Substrate RBP4-Retinol Can Activate RA Direct Targets in Epithelial Cells

**Figure 2:**
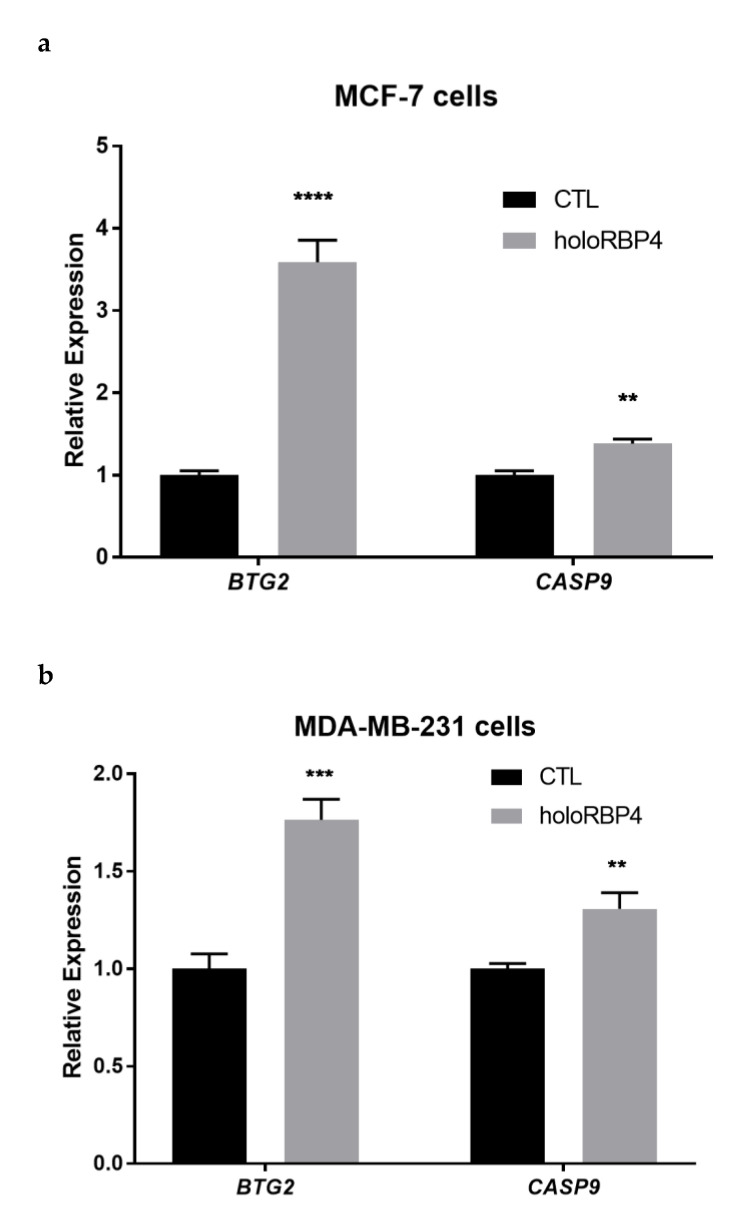

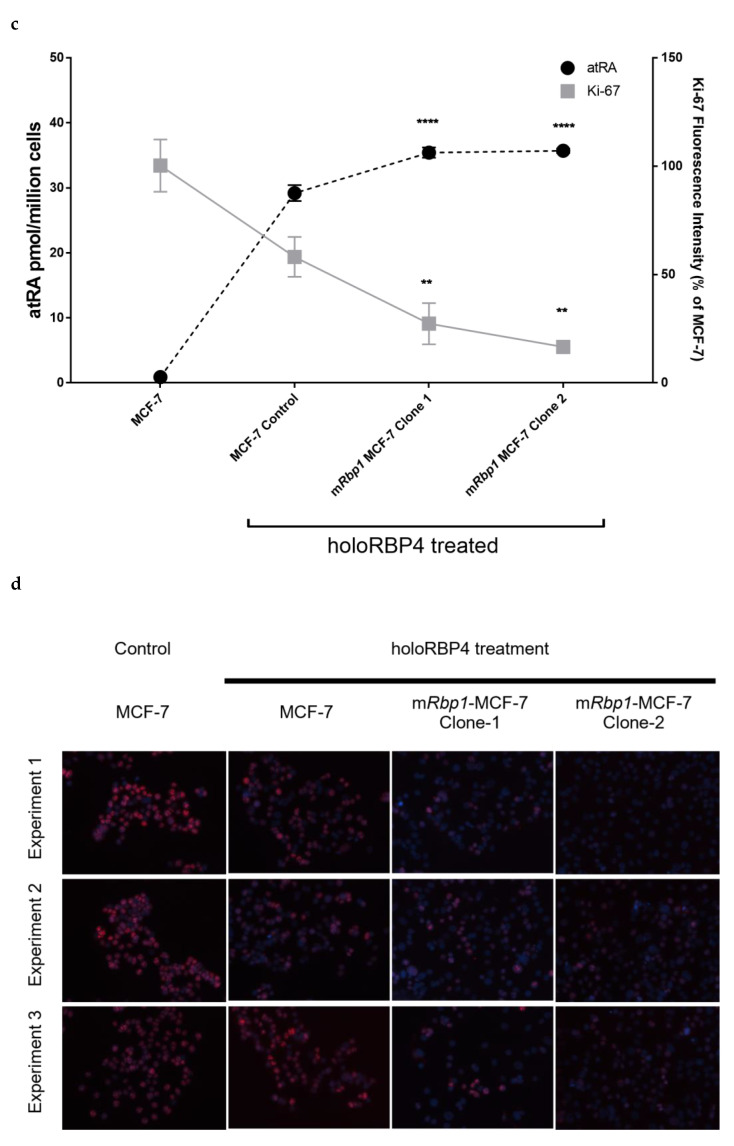
atRA produced from physiological substrate holoRBP4 stimulated transcriptional targets; atRA and RBP1 expression inhibited proliferation. Physiological substrate holoRBP4 stimulated RA anti-proliferative direct target genes *BTG2* and *CASP9* expression in (**a**) MCF-7 cell lines and (**b**) MDA-MB-231 cell lines. MCF-7 cell line were treated with holoRBP4 for 18 h. *BTG2* and *CASP9* gene expression was quantified by QPCR normalized to beta-actin. Data were mean ± SD, *n* = 3. (**c**) Overexpression of RBP1 in MCF-7 cells inhibited cell proliferation. Stable overexpression m*Rbp1* clones and parent MCF-7 cells were treated with holoRBP4 for 5 days. atRA was quantified with LC-MRM^3^ (left *y*-axis) and Ki-67 staining was quantified by ImageJ normalized to DAPI staining (right *y*-axis). Data were mean ± SD, *n* = 3. (**d**) Overexpression of RBP1 in MCF-7 cells had less Ki-67 positive cells. Stable overexpression m*Rbp1* clones and parent MCF-7 cells were treated with holoRBP4 for 5 days. Cells were fixed and stained for Ki-67 (red). Ki-67 signal intensity were normalized to DAPI (blue) staining. Pictures were shown as merged. Ki-67 staining positive shown as purple. Ki-67 staining negative shown as blue. Statistical analysis: Figure 2a,b, unpaired parametric two-tailed student t test. ** *p* ≤ 0.01; *** *p* ≤ 0.001; **** *p* ≤ 0.0001. Figure 2c, ordinary one-way ANOVA analysis corrected for multiple comparisons using Dunnett’s test (with comparison to the control). The ANOVA results were: atRA, F(3, 8) = 1488, **** *p* < 0.0001, Ki-67, F(3, 8) = 52.19, ** *p* < 0.0001.

To determine the impact of modulating cellular atRA, we quantified the expression of known transcriptional targets of atRA, B-cell translocation gene, member 2 (*BTG2*) and caspase 9 (*CASP9*), using QPCR in MCF-7 and MDA-MB-231 cells that were treated with 2 µM holoRBP4 for 18 h (Figure 2a,b). We chose *BTG2* and *CASP9* because they have previously been characterized as direct targets of atRA in MCF-7 cells [53,54]. *CASP9* is a component of the apoptotic response and *BTG2* inhibits cell cycle progression; both have been characterized as contributing to atRA’s anti-proliferative effect in MCF-7 cells [53,54]. Cells that were treated with holoRBP4 had greater atRA (data not shown, similar to atRA shown in Figure 2c) and increased expression of *BTG2* and *CASP9* in MCF-7 (Figure 2a) and MDA-MB-231 (Figure 2b) cells.

### 3.4. Overexpression of RBP1 and atRA Inhibits Cell Proliferation in MCF-7 Cells

In addition to the expression of anti-proliferative direct transcriptional targets of atRA, *BTG2* and *CASP9*, we used Ki-67 staining as a marker for proliferating cells to investigate the functional impact of atRA and RBP1 expression. We quantified proliferation in tumorigenic MCF-7 cells, as well as in two independent clones of MCF-7 that overexpress RBP1 and treated with the substrate for atRA biosynthesis, holoRBP4 (Figure 2c,d). MCF-7 cells provided with holoRBP4 produced more atRA and had reduced proliferation compared to control MCF-7 cells that did not provide holoRBP4 (Figure 2c,d). MCF-7 clones that overexpressed RBP1 had more atRA production and had further reduced proliferation compared to control MCF-7 cells treated with and without holoRBP4 (Figure 2c,d).

### 3.5. RBP1 Expression and atRA Inhibited Collagen Deposition in Cell Culture

**Figure 3:**
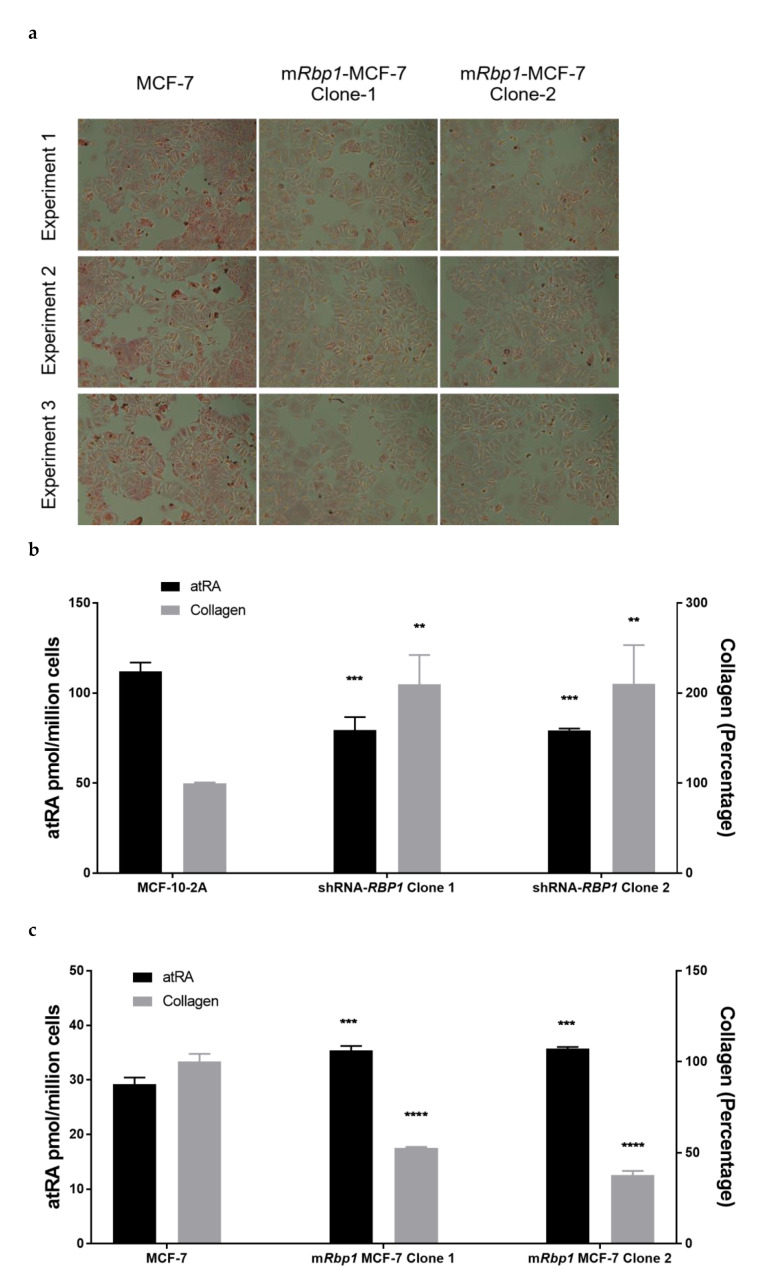

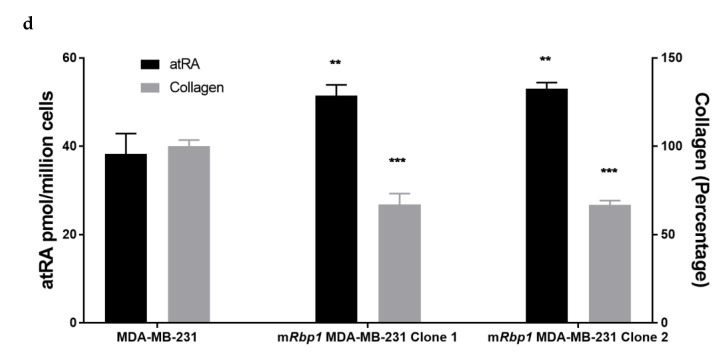
RBP1 expression and atRA inhibited collagen deposition in cell culture. (**a**) Overexpression of RBP1 in MCF-7 cells reduced collagen deposition. Stable overexpression *mRbp1* clones and parent MCF-7 cells were treated with holoRBP4. Cells were seeded to 12 well plates. Culture media was removed after 18 h incubation. Cells were fixed and stained by picrosirius red as described in Methods section. Collagen deposition in 2 independent *RBP1* stable overexpression clones was less than parent MCF-7 cells. (**b**) Knockdown of RBP1 expression in normal MCF-10-2A resulted in an increase in collagen deposition. Stable shRNA-*RBP1* knock down clones and parent MCF-10-2A cells were treated with holoRBP4. atRA (left *y*-axis) and collagen deposition (right *y*-axis). Data are mean ± SD, *n* = 3. Overexpression of RBP1 in MCF-7 cells (**c**) and MDA-MB-231 cells (**d**) reduced collagen deposition. Stable overexpression m*Rbp1* clones and parent cells were treated with holoRBP4. atRA (left *y*-axis) and collagen deposition (right *y*-axis). Data were mean ± SD, *n* = 3. atRA quantified with LC-MRM^3^ (left *y*-axis) and collagen deposition quantified by Picrosirius red staining (right *y*-axis). Statistical analysis: Figure 3b–d, ordinary one-way ANOVA analysis corrected for multiple comparisons using Dunnett’s test (with comparison to the respective control in each panel). The ANOVA result summary were as follows: Figure 3b, atRA, F(2, 6) = 41.34, *** *p* = 0.0003, Collagen, F(2, 6) = 12.65, ** *p* = 0.0070; Figure 3c, atRA, F(2, 6) = 55.32, *** *p* = 0.0001, Collagen, F(2, 6) = 397.8, **** *p* < 0.0001; Figure 3d, atRA, F(2, 6) = 20.13, ** *p* = 0.0022, Collagen, F(2, 6) = 58.68, *** *p* = 0.0001.

To further quantify the functional impact of atRA and RBP1 expression, we quantified collagen by picrosirius red staining (Figure 3). We measured collagen because excess collagen was observed in the *RBP1^-/-^* mammary [31] and atRA has been shown to reduce collagen in cells in culture [55]. Picrosirius red staining showed that tumorigenic MCF-7 clones that overexpress RBP1 had repeatably less collagen than control MCF-7 cells (Figure 3a). Further correlation showed that collagen staining was inversely proportional to atRA levels (Figure 3b–d). In normal MCF-10-2A cells, knock-down of RBP1 expression had reduced atRA production and increased collagen deposition (Figure 2b). Tumorigenic MCF-7 and MDA-MB-231 clones that overexpress RBP1 had increased atRA production and reduced collagen deposition (Figure 3c,d).

### 3.6. Tumor Microenvironmental Factors in Culture Condition Alter RBP1 Expression and atRA Homeostasis in Cell Lines

**Figure 4:**
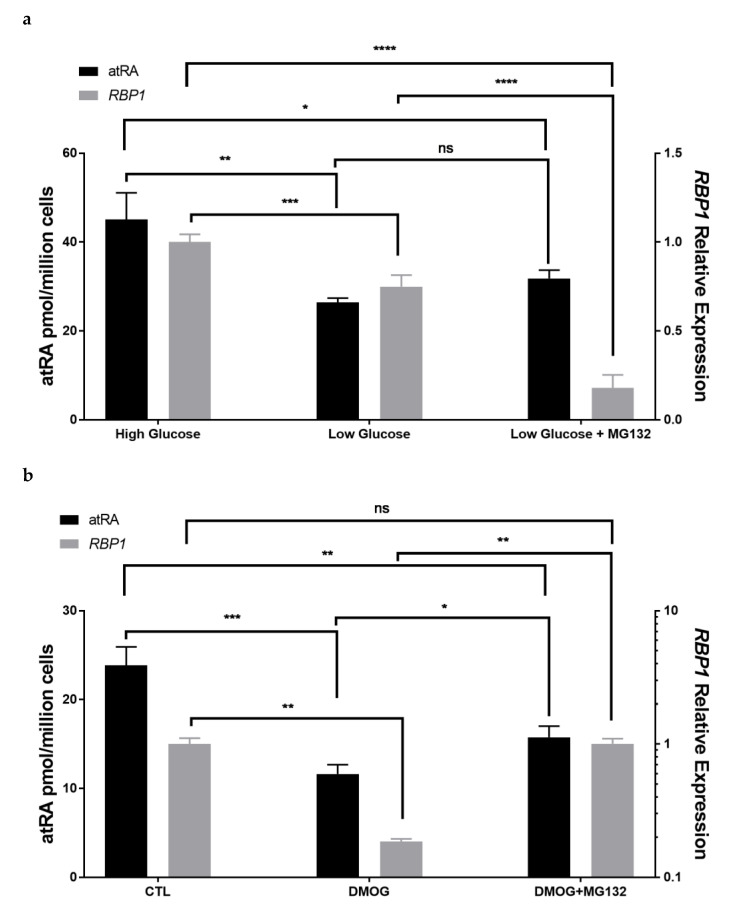

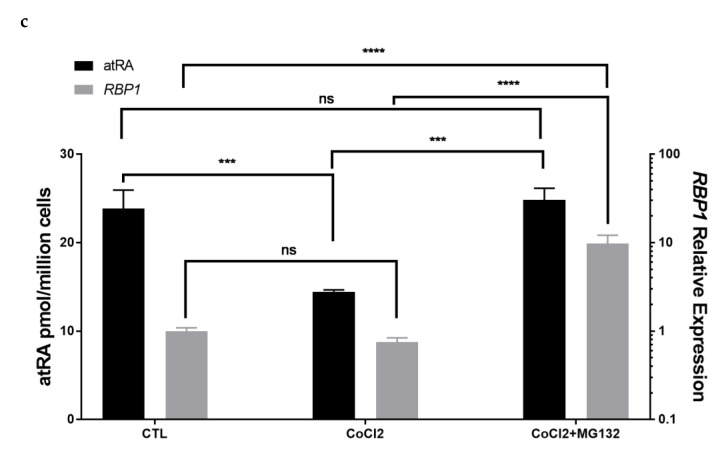
Microenvironmental factors altered *RBP1* expression and atRA production. (**a**) Lower glucose decreased atRA biosynthesis and reduced *RBP1* gene expression in MCF-10-2A cells. MG132 treatment after low glucose increased atRA production but not the RBP1 gene expression. MCF-10-2A cells were culture in low glucose (1 g/L) for 48 h and MG132 (1 µM) was added at the last 24 h. atRA (left *y*-axis) and RBP1 gene expression (right *y*-axis). Data were mean ± SD, *n* = 3. (**b**) DMOG treatment decreased both atRA production and Rbp1 gene expression in MCF-10-2A cells. MG132 treatment reversed the DMOG effect. MCF-10-2A cells were culture with DMOG (1 mM) for 48 h and MG132 (1 µM) was added at the last 24 h. atRA (left *y*-axis) and RBP1 gene expression (right *y*-axis). Data were mean ± SD, *n* = 3. (**c**) CoCl_2_ treatment decreased both atRA production and Rbp1 gene expression in MCF-10-2A cells. MG132 treatment reversed the CoCl_2_ effect. MCF-10-2A cells were culture with CoCl_2_ (150 µM) for 48 h and MG132 (1 µM) was added at the last 24 h. atRA (left *y*-axis) and RBP1 gene expression (right *y*-axis). Data were mean ± SD, *n* = 3. atRA was quantified with LC-MRM^3^ (left *y*-axis) and RBP1 gene expression was quantified by QPCR normalized to beta-actin (right *y*-axis). Statistical analysis: Figure 4a–c, ordinary one-way ANOVA analysis corrected for multiple comparisons using Tukey’s multiple comparison test (compare each with every other). The ANOVA result summary were as follows: Figure 4a, atRA, F(2, 6) = 20.2, *p* = 0.0022, *RBP1*, F(2, 9) = 184.9, *p* < 0.0001; Figure 4b, atRA, F(2, 6) = 49.57, *p* = 0.0002, *RBP1*, F(2, 3) = 65.84, *p* = 0.0033; Figure 4c, atRA, F(2, 6) = 48.86, *p* = 0.0002, *RBP1*, F(2, 7) = 79.25, *p* < 0.0001. Statistical significance for pairwise comparisons is notated as follows: ns *p* > 0.05; * *p* ≤ 0.05; ** *p* ≤ 0.01; *** *p* ≤ 0.001; **** *p* ≤ 0.0001.

Tumor microenvironments often have low glucose and/or hypoxia [56]. To investigate if microenvironmental factors may cause or contribute to reduced RBP1 expression and reduced atRA, we quantified the impact of low glucose or hypoxia in MCF-10-2A cells (Figure 4).

RBP1 expression and atRA production were quantified in MCF10 cells cultured with low glucose (1.0 g/L) and compared to high glucose (4.5 g/L), where the high glucose condition was the level of glucose typically present in the culture of MCF-10-2A cells. Low glucose reduced RBP1 expression and atRA production compared to high glucose (Figure 4a). To see if the reduction in RBP1 expression was due to increased protein degradation, we treated with MG132 to block protein degradation. MG132 is a potent, reversible and cell permeable proteasome inhibitor that has previously been shown to modulate RBP1 levels in MCF-7 cells [39]. Blocking protein degradation did not restore RBP1 expression (Figure 4a).

We used two different agents to effect hypoxia in MCF-10-2A cells, DMOG and CoCl_2_. DMOG is cell-permeable and a competitive inhibitor of HIF-hydroxylases, thereby enabling stabilization and heterodimerization of HIF-1α, which has been extensively applied as a tool to mimic hypoxia [47]. CoCl2 is also a commonly used model to mimic hypoxia, where CoCl2-induced chemical hypoxia stabilizes hypoxia inducible factors 1α and 2α under normoxic conditions [48]. Both DMOG and CoCl_2_ have been used in MCF-7 and MDA-MB-231 cells [40,41,42,43,44,45,46]. Both agents reduced RBP1 expression and atRA production compared to control (Figure 4b,c). Treatment with MG132 to block protein degradation increased RBP1 expression and atRA in both DMOG (Figure 4b) and CoCl_2_ (Figure 4c) treated MCF-10-2A cells.

### 3.7. Therapeutic Treatment Increases RBP1 Expression and atRA Production

**Figure 5:**
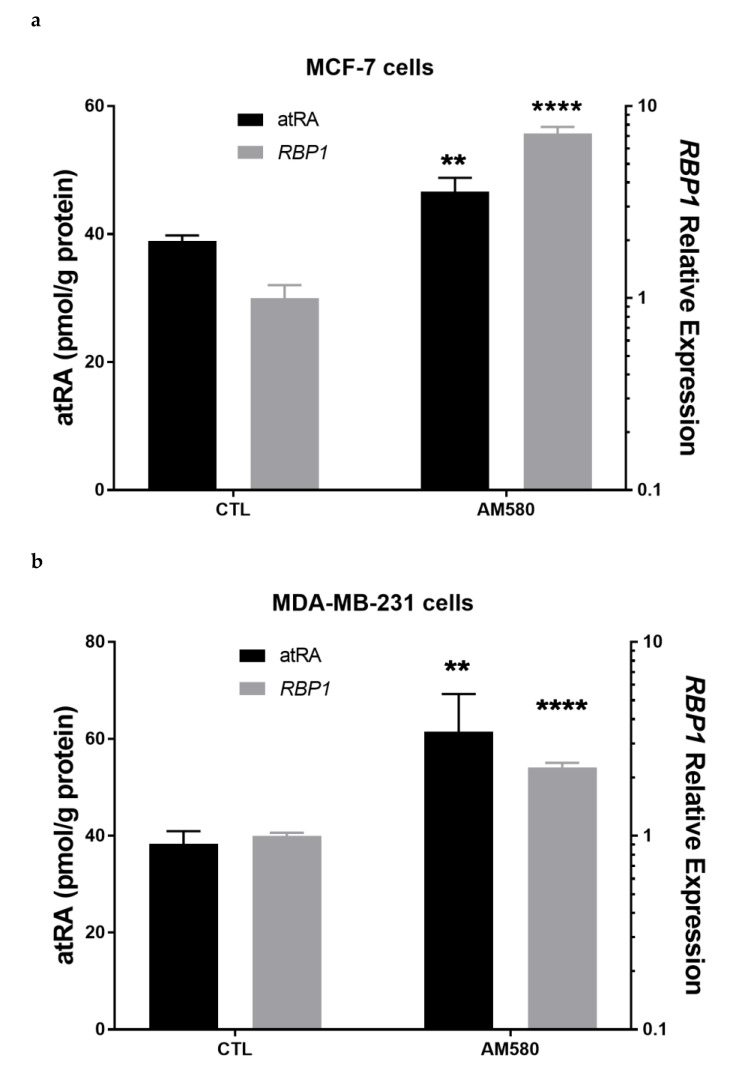

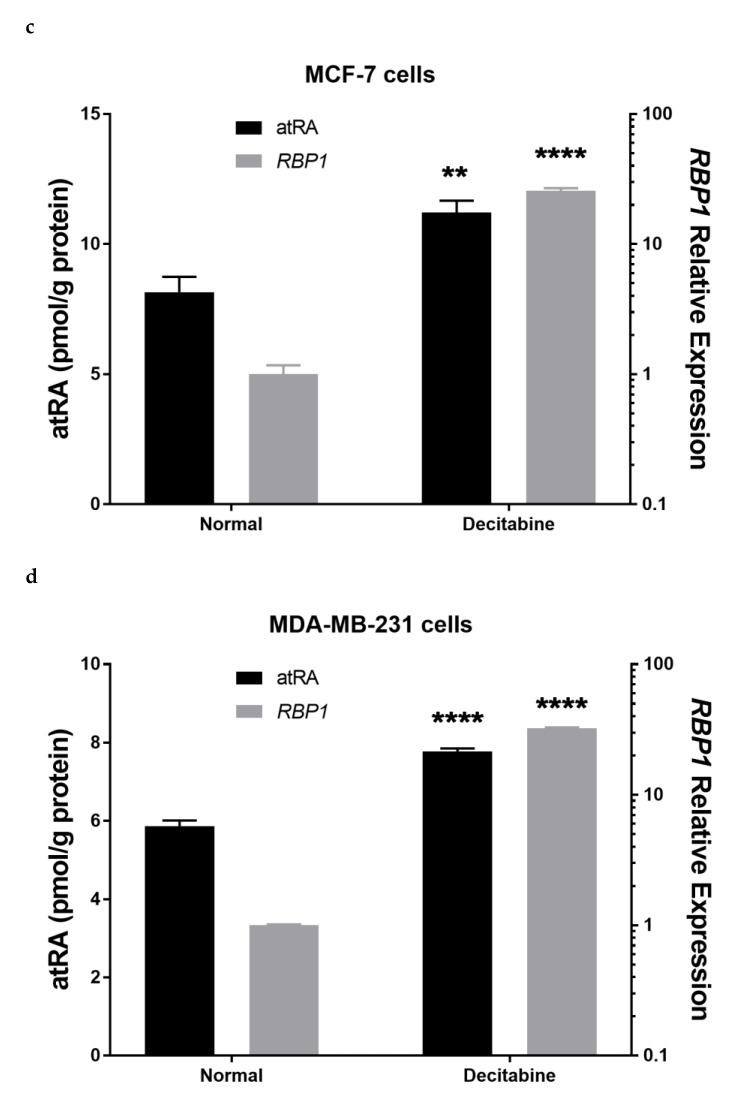
Therapeutic treatment increased RBP1 expression and atRA production. (**a**) MCF-7 cell and (**b**) MDA-MB-231 cell were treated with 200 nM RARα agonist AM580 for 48 h followed by holoRBP4 for 3 h. atRA (left *y*-axis) and *RBP1* gene expression (right *y*-axis). Both *RBP1* gene expression and atRA production were increased. (**c**) MCF-7 cell and (**d**) MDA-MB-231 cell treated with Decitabine increased RBP1 expression and atRA production. MCF-7 cells were treated with 10 µM Decitabine for 3 days followed with holoRBP4 for 3 h. (left *y*-axis) and *RBP1* gene expression (right *y*-axis). Data were mean ± SD, *n* = 3. atRA was quantified with LC-MRM^3^ (left *y*-axis) and *RBP1* gene expression was quantified by QPCR normalized to beta-actin (right *y*-axis). Statistical analysis: Figure 5a–d, unpaired parametric two-tailed student *t* test. ** *p* ≤ 0.01; **** *p* ≤ 0.0001.

AM580 is an RARα agonist that has been shown to increase the expression of RBP1 [49,50]. To determine if treatment with AM580 could increase RBP1 expression and atRA production, we treated MCF-7 and MDA-MB-231 cells with 200 nM AM580. We observed an increase in RBP1 expression and atRA production in both MCF-7 (Figure 5a) and MDA-MB-231 (Figure 5b) cells.

As loss of RBP1 expression in cancer has been reported to be mainly due to hypermethylation [18,19,39,57], we treated with a demethylating agent, decitabine [18]. Decitabine inactivates DNA methyltransferase 1 in replicating cells, effectively reducing methylation, including in MCF-7 and MDA-MB-231 cells [38,58]. Treatment with 10 µM decitabine resulted in increased RBP1 expression and atRA production in both MCF-7 (Figure 5c) and MDA-MB-231 (Figure 5d) cells.

### 3.8. Epithelial atRA Production Capacity Impacts Neighboring Fibroblast Cell atRA Supply

**Figure 6:**
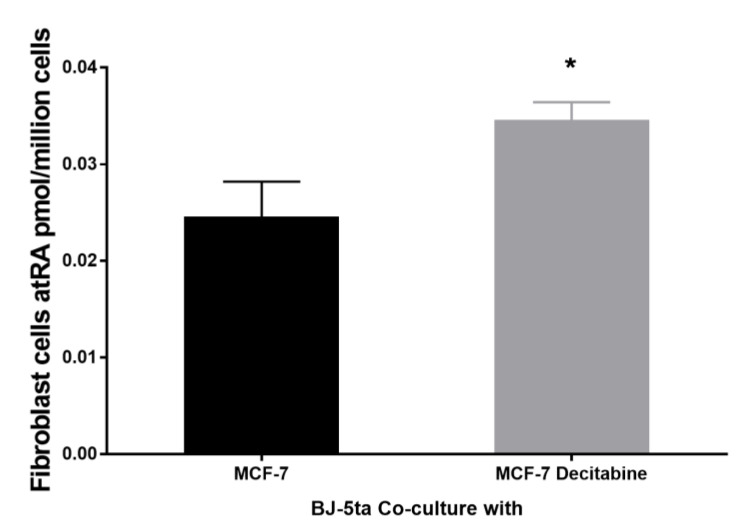
Epithelial cell atRA production impacted neighboring fibroblast cell atRA content. Epithelial cell atRA production impacted neighboring fibroblast cell atRA content. Fibroblast cell BJ-5ta were co-cultured with MCF-7 and Decitabine treated MCF-7 cells for 18 h then treated with holoRBP4 for 3 h. Cells were detached and separated. Fibroblast cell atRA was quantified with LC-MRM^3^ and normalized to cell number. Data are mean ± SD, *n* = 3. Statistical analysis: Figure 6, unpaired parametric two-tailed student *t* test. * *p* ≤ 0.05.

Fibroblast dysfunction in the tumor microenvironment has been hypothesized to be impacted by epithelial cell dysfunction [59,60]. Monoculture of MCF10-2A epithelial cells, MCF-7 epithelial cells, and BJ-5ta fibroblasts show that normal epithelial cells have greater levels of cellular atRA than fibroblasts. Normal MCF-10-2A epithelial cells in monoculture had cellular levels of atRA of 0.23 ± 0.02 pmol/million cells as compared to (normal) BJ-5ta fibroblasts that had only 56% of that at 0.13 ± 0.001 pmol/million cells of cellular atRA. Tumorigenic MCF-7 epithelial cells in monoculture also had reduced atRA, compared to normal MCF-10-2A, with cellular levels of 0.14 ± 0.05 pmol/million cells atRA. To determine if neighboring fibroblast cells are impacted by the ability of epithelial cells to produce atRA, we co-cultured BJ-5ta fibroblasts with epithelial cells, followed by isolation of the fibroblast cells and quantification of their cellular atRA levels. Fibroblasts co-cultured with tumorigenic MCF-7 epithelial cells had 57% less cellular atRA than fibroblast co-cultured with normal MCF-10-2A cells, with 0.065 ± 0.005 pmol/million cells and 0.151 ± 0.032 pmol/million cells cellular atRA, respectively (mean ± SD, *n* = 3, *p* = 0.0112). To mimic a potential therapeutic effect, we co-cultured fibroblasts with MCF-7 epithelial cells treated with decitabine, which we showed to increase RBP1 expression and atRA in MCF-7 cells (Figure 5) and compared those to fibroblast co-cultured with MCF-7 cells (Figure 6). Fibroblasts co-cultured with MCF-7 epithelial cells treated with decitabine had increased cellular atRA compared to the cellular atRA of fibroblasts co-cultured with control MCF-7 cells (Figure 6).

## 4. Discussion

### 4.1. RBP1 and atRA Homeostasis

RBP1 expression and atRA have a direct relationship in both human mammary tissue and in human mammary epithelial cell lines. Tumorigenic tissue and cell lines had lower RBP1 expression and lower atRA. Manipulation of atRA through knockdown of RBP1 in normal cell lines and overexpression of RBP1 in tumorigenic cell lines resulted in a corresponding decrease and increase in atRA production, respectively (Figure 1). This work provides direct quantification of atRA in tumors and cell lines and the effect of modulating RBP1 expression using rigorous analytical methods [12,13,27,28]. Many previous studies inferred atRA levels based upon pathway or target gene expression due to the technical challenges of atRA quantification.

The direct relationship between RBP1 expression and atRA is consistent with reduced atRA levels in the *Rbp1^-/-^* mammary [31]. *Rbp1^-/-^* hyperplastic tissue and human hyperplastic tissue with reduced RBP1 have a similar degree of atRA reduction: ~35–40% [30,31]. We have also reported reduced atRA in other *Rbp1^-/-^* tissues, including heart, lung, endometrium, testis, and liver [27,28,29,30,31]. A direct relationship between RBP1 expression and atRA may indicate that other cancers, in addition to DCA, with reduced RBP1 also have reduced atRA including other types of breast, prostate, lung, colon and rectal, melanoma, bladder, non-Hodgkin lymphoma, leukemia, endometrial, and pancreatic cancer [17,18,19,20,21,22,23,24,25,26]. The direct relationship between RBP1 and atRA may also be relevant to other human diseases with reduced RBP1, including endometriosis [30] and heart failure [61].

### 4.2. RBP1 and atRA Impact the Microenvironment

We focused on epithelial cells because approximately 90% of human tumors originate from epithelial cells [60]. Epithelial cells express RBP1 and produce atRA. Because atRA is a master regulator of proliferation, differentiation, and apoptosis, [3,4,7,14,53,54], we hypothesized that it would impact cellular functions that contribute to a dysfunctional microenvironment. RBP1 expression and atRA levels had an inverse relationship with both proliferation and collagen deposition. This was consistent with the in vivo phenotype of the *Rbp1^-/-^* mammary, which had reduced atRA, hyperplasia of the epithelial cells, and increased collagen [31].

In addition to showing that RBP1 expression and atRA levels impacted epithelial cell proliferation and collagen, we showed that epithelial cell RBP1 expression and the ability of epithelial cells to produce atRA impacts the atRA content of neighboring fibroblast cells in a co-culture model (Figure 6). We based our co-culture system on work by Lisanti and co-workers who used epithelial cell-fibroblast co-culture systems to mimic the in vivo microenvironment to study the co-evolution of tumor-stroma defects, and the effect of various factors in the microenvironment [62,63,64]. The increase in fibroblast cell atRA was proportional to the capacity of neighboring epithelial cells to produce atRA. Other studies have shown that atRA biosynthesis in one cell type can influence neighboring cells and that atRA biosynthesis and signaling can influence microenvironmental events, such as cell fate, cell homing, cell migration, and protein secretion [65,66,67,68,69,70]. As retinoids are easily oxidized, paracrine transport of atRA likely involves a chaperone and/or transport vehicle, such as an extracellular vesicle or exosome [71,72,73].

Fibroblasts in the microenvironment become dysfunctional in cancer due to, in part, microenvironmental cues from neighboring cells [59]. In addition to RBP1 and atRA being key regulators of mammary epithelial cells, atRA may also be a microenvironmental signal for maintaining mammary tissue homeostasis, including fibroblast cells. Epithelial cell dysfunction can lead to fibroblast cell accumulation and an activated phenotype that secretes excess collagen [74,75]. *Rbp1^-/-^* mammary had hyperplasia of neighboring fibroblast cells, as well as hyperplasia of the epithelial cells and increased collagen [31]. Fibrotic defects resulting from excess collagen accumulation are associated with poor prognosis and are linked to a greater than four-fold increased risk of cancer progression, invasion, and recurrence [74,76,77,78].

### 4.3. Microenvironmental Factors Impact RBP1 Expression

To understand factors that could contribute to the reduction in RBP1 and the reduction in atRA, we investigated cellular conditions that have been reported to be present in tumor microenvironments, including low glucose and hypoxia [56]. Low glucose and hypoxia reduced RBP1 expression and atRA. Protein degradation contributed to the hypoxia-induced reduction in RBP1 as blocking of protein degradation by MG132 recovered RBP1 expression and atRA. Protein degradation is important in cancer and has numerous mechanism by which it is regulated [79]. Understanding regulation of RBP1 protein levels would be of interest in future studies. Reduced RBP1 expression by low glucose was not restored by blocking protein degradation and must occur via a different mechanism. These microenvironmental factors, including glucose and hypoxia, could either contribute to the initial decrease in RBP1 and atRA or could contribute to the further, progressive decline in RBP1 expression and atRA levels as a function of tumorigenesis, which was observed in human cell lines and cancer (Figure 1).

### 4.4. Therapeutic Potential for Restoring RBP1 Expression and Endogenous atRA Production

Consistent with the direct relationship between RBP1 expression and atRA (Figure 1), we observed that therapeutics that raised RBP1 expression also raised atRA in epithelial cells (Figure 5). This restoration in epithelial cells also resulted in increased atRA content in neighboring fibroblast cells (Figure 6). AM580 is an RARα agonist that has previously shown to reduce tumor size and inhibit proliferation [49,50]. Decitabine is a demethylating agent that reduces the hypermethylation that represses RBP1 expression in cancer [80]. Previous studies have shown that targeted epigenetic agents that increase RBP1 also increase atRA [32,81]. Restoring atRA through increasing endogenous atRA production facilitated by RBP1 has been suggested as a therapeutic strategy [32]. This work establishes a direct relationship between RBP1 expression and atRA which is maintained when RBP1 expression is restored therapeutically. In addition to breast cancer, a number of other diseases show reduced RBP1 expression and reduced atRA including other types of cancer, endometriosis, and heart failure. Diseases with reduced RBP1 could benefit from therapeutics that restore RBP1 expression and endogenous atRA. These findings reported here likely extend to other diseases with reduced RBP1, however direct testing is required.

### 4.5. Limitations and Future Opportunities

Our study had several limitations that present opportunities for future experimentation. While we observed a direct relationship between RBP1 expression and atRA levels in human tumors, we did not perform a statistical correlation. A future study with larger numbers of samples would enable the determination of the statistical strength of this direct relationship. Our gene expression data was only normalized to b-actin; it would be strengthened by normalizing to multiple genes. We did not measure the protein levels of RBP1 here, we used only the mRNA expression of RBP1. In previous studies the mRNA of RBP1 has been shown to agree with protein levels of RBP1 [57]. We are currently validating a liquid chromatography-tandem mass spectrometry-based targeted quantification method for RBP1 protein, which will enable direct quantitation of RBP1 in future studies with the improved sensitivity that is needed for biological samples, compared to our initial native mass spectrometry-based method development [82]. We also did not measure the methylation status of RBP1, but literature as well as ours, and other decitabine experiments, indicate it as a contributing factor in the reduction of RBP1 expression [18,19,39,57]. It would be interesting to further interrogate how the effect of variable levels of methylation that lead to reduced RBP1 expression impact atRA levels. Additionally, we relied on the literature for hypoxia conditions [40,41,42,43,44,45,46] and did not directly measure levels of hypoxia. A future study investigating the relationship between hypoxia and atRA and RBP1 would be of interest.

## Data Availability

Not applicable.
